# Management of Acute Lower Gastrointestinal Bleeding: A Survey to Assess Adherence to Guidelines Across the United Kingdom and Ireland

**DOI:** 10.7759/cureus.25273

**Published:** 2022-05-24

**Authors:** Muhammad Fahad ullah, Sofian Youssef, Nikhil Kulkarni, Milind Rao

**Affiliations:** 1 General Surgery, Pilgrim Hospital, Boston, GBR; 2 General Surgery, Lincoln County Hospital, Lincoln, GBR

**Keywords:** bsg guidelines for lower gi bleed, oakland score, colonoscopy, ct angiogram, lower gi bleed

## Abstract

Introduction: Acute gastrointestinal bleeding (GIB) is a common surgical problem requiring hospitalization in the United Kingdom (UK) and Ireland. The first UK lower gastrointestinal bleeding (LGIB) management guidelines were published in 2019 by the British Society of Gastroenterology (BSG). We aimed to evaluate self-reported adherence to BSG clinical guidance (CG) within the UK and Ireland.

Methods and materials: A Questionnaire was designed based on LGIB BSG CG 2019 using Google Forms (Google LLC, Mountain View, CA). This was distributed to surgical consultants and senior surgical practitioners (specialists, Trust grade registrars, and specialist registrars) across different centers in the UK and Ireland over four weeks (13th March to 5th April 2021). Data were analyzed using Statistical Package for Social Sciences (SPSS) version 27 (IBM Corp., Armonk, NY, USA).

Results: A total of 64 responses were recorded from 18 different centers in the UK and Ireland. The ratio of consultants and registrars was almost the same (34:30, 53.1%:46.9%). The majority of respondents were from colorectal surgery (65.6%, n=42) followed by general surgeons (23.4%, n=15). A total of 41 respondents (64.1%) admitted that BSG CG 2019 were practically applicable at their center. Approximately 75% of respondents did not use or were unaware of the Shock index or Oakland score to stratify patients. That translated into 59% opting to admit patients with a minor bleed. Around 36% wanted to perform a CT angiogram for a stable major bleed, while 37% were unaware of the interventional radiology (IR) referral pathway.

Conclusion: There is patchy adherence to the guidelines leading to significant variations in LGIB management practice and avoidable admissions.

## Introduction

Acute gastrointestinal bleeding (GIB) is a common surgical problem, accounting for approximately 85,000 cases every year in the United Kingdom (UK), which is approximately one GIB every six minutes [1]. Lower gastrointestinal bleeding (LGIB) has an estimated annual incidence of up to 87/10000 [2], with an estimated admission rate of 33 to 87/100000 [3]. The overall mortality for such patients ranges from 3.4% up to 17.8% for inpatients [4]. The most common cause of LGIB in the UK is diverticulitis [2,4]. Most acute LGIB resolves spontaneously, not requiring hospital intervention, and can therefore be managed in an outpatient setting [5]. Very few suffer from severe, persistent hemorrhage and rebleeding [6]. Hence, a risk stratification strategy was needed to avoid unnecessary admissions and investigations, and to identify patients requiring hospital admission for intervention.

Over the last decade, greater focus has been placed on the adoption of uniform clinical practice based on the best available, high-quality evidence, synthesized in the form of guidelines. In 2015, the National Confidential Enquiry into Patient Outcome and Death (NCEPOD) report revealed increasing incidence, morbidity, and mortality associated with GIB, heeding the development of clinical guidelines to improve such outcomes [1]. The National Institute for Health and Care Excellence (NICE) has previously published comprehensive guidelines for the management of upper gastrointestinal bleeding (UGIB) [7]. However, there were no equivalent guidelines for the management of LGIB until 2019, when the British Society of Gastroenterology clinical guidelines (BSG CG 2019) were published [8].

The proposed BSG guidelines provide a tool for clinicians to stratify the patients who can be safely discharged home (low risk) for further investigation and provide a road map for further management of those who need hospital-based treatment. Evidence suggests that clinical guidelines improve the quality of care [9] but non-compliance is as high as 70% [10]. To date, there has been no formal evaluation of the adherence to and awareness of BSG CG 2019 among clinicians. This survey, therefore, aimed to assess the awareness and adherence of surgical trainees and consultants to the BSG CG 2019 LGIB management guidelines, two years following its publication.

## Materials and methods

Study design

This was a cross-sectional exploratory study, evaluating surgeons' reported adherence to BSG CG 2019 [8]. Inclusion criteria comprised the UK and Ireland surgical registrars of all grades, and consultants involved in the care of LGIB patients.

Survey

An anonymized 14-point questionnaire was distributed nationally via surgical registrars (trainees and non-trainees) and surgical consultant groups working at hospitals that deal with patients presenting with symptoms of lower GI bleed. Data were gathered between 15th March to 6th April 2021 using WhatsApp, Email, and Social media platforms such as Twitter and Facebook. Google Forms (Google LLC, Mountain View, CA) was used to collect responses, which automatically recorded data in an encrypted spreadsheet by Google Sheets (Google LLC, Mountain View, CA). Questions were formed based on key recommendations stipulated in LGIB 2019 [8].

Analysis

Data were analyzed using Statistical Package for Social Sciences (SPSS) version 27 (IBM Corp., Armonk, NY, USA). Data were analyzed using descriptive statistics; categorical variables are presented as absolute figures (percentages), and ordinal Likert (1-5) scale outcome variables are presented as median (interquartile [IQR] range, Q1-Q3).

## Results

A total of 64 responses were recorded from 18 different centers in the UK and Ireland, 34 (53.1%) of which were from registrars and 30 (46.9%) from consultants. Two-thirds (n=42, 65.6%) reported their main specialty of practice as colorectal surgery, 15 (23.4%) were general surgeons, and seven (10.9%) were upper GI surgeons. A total of 41 respondents (64.1%) thought BSG CG 2019 was practically applicable at their center, 13 (20.3%) did not, and 10 (15.6%) reported they were unsure. The results are summarised in Table 1.

**Table 1:** The 14-point electronic survey questions, with answer options and the absolute number of responses (%), median (IQR, Q1-Q3). Google Forms was used to collect responses. +/-: Plus/minus, GI: Gastrointestinal, IR: Interventional radiology, IQR: Interquartile range

Question	Answer Options	Frequency	
		Number	%
What is your main hospital site?	-		
What county is your hospital situated in?	-		
Define your role, please	Consultant	30	46.9
	Registrar	34	53.1
What is your main specialty of practice?	Colorectal	42	65.6
	Upper GI	7	10.9
	General	15	23.4
Do you calculate the Shock index?	Yes	14	21.9
	No	44	68.8
	Unsure	6	9.4
Do you calculate the OAKLAND score?	Yes	11	17.5
	No	48	76.2
	Unsure	4	6.3
How do you manage minor stable lower GI bleed?	Admit for observation	19	29.7
	Admit for observation if they are on anticoagulation	18	28.1
	Admit for observation and CT angiography	1	1.6
	Discharge/Conservative management and outpatient investigations	26	40.6
How do you manage stable major lower GI bleed?	Admit for lower GI endoscopy on the next list	21	32.8
	Admit for conservative management and outpatient endoscopy	20	31.3
	Admit for CT angiography	23	35.9
How do you manage unstable lower GI bleeds?	Resuscitation+/- Urgent Lower GI endoscopy +/- Surgical intervention	9	14.1
	Resuscitation +/- Urgent CT angiogram +/- IR embolisation +/- Surgical intervention	55	85.9
	Resuscitation to stabilize +/- Surgical intervention	0	0
Do you have onsite interventional radiology (IR) facilities?	Yes	43	67.2
	No	21	32.8
	Unsure	0	0
If yes, what are the working hours of your IR facility?	24 hours a day	27	57.4
	Normal working hours	20	42.6
If you do not have onsite interventional radiology (IR) facility, do you have a referral pathway for IR embolization?	Yes	33	61.1
	No	15	27.8
	Unsure	6	11.1
How easy do you find it to refer a patient for IR embolization at night?	Likert Scale (1-5) Extremely Difficult – Extremely Easy	Median 3 (IQR 2 - 4)	
Do you think the proposed algorithm is practically applicable in your center?	Yes	41	64.1
	No	13	20.3
	Unsure	10	15.6

Lower gastrointestinal bleeding (LGIB) guidelines

Shock Index & Oakland Score

A total of 44 (68.8%) respondents reported they do not typically calculate the Shock index, 14 (21.9%) reported they do, and six (9.4%) were unsure. A total of 48 (76.2%) respondents reported they do not typically calculate the Oakland score, 11 (17.5%) reported they do, and four were unsure.

Management of Minor Stable Lower GI Bleeds

A total of 26 (40.6%) reported they would discharge minor stable lower GI bleeds with conservative management and investigate them as outpatients. Of the respondents, 37 (57.8%) reported they would admit patients with minor bleeds, 19 (29.7%) reported they would admit such patients for observation, and a further 18 (28.1%) reported they would admit patients for observation if they were anticoagulated. Only one (1.6%) reported they would admit for observation and investigation with CT angiography.

Management of Major Stable Lower GI Bleeds

Twenty-three respondents reported they would carry out a CT angiogram after admission for a major stable bleed. A further 21 (32.8%) reported they would admit for urgent lower GI endoscopy, and the remaining 20 (31.3%) reported they would admit for conservative management and investigation of such patients as outpatients.

Management of Major Unstable Lower GI Bleeds

Of the respondents, 55 (85.6%) reported they would resuscitate major unstable lower GI bleeds with or without urgent CT angiography, IR embolization, and surgical intervention, as indicated. The remaining nine (14.1%) respondents reported they would resuscitate with or without urgent lower GI endoscopy and surgical intervention, as indicated.

Management with Interventional Radiology

Forty-three (67.2%) of the respondents reported having onsite IR facilities. For 27 (57.4%), the IR was accessible 24 hours a day and the remaining 20 (42.6%) could access it only during normal working hours. Of those with onsite IR facilities, 33 (61.1%) had a referral pathway for embolization, 15 (27.8%) did not, and six (11.1%) were unsure. On a Likert scale with 1 being extremely difficult to 5 being extremely easy), the median reported ease of referring patients for IR embolization at night was 3 (interquartile range, 2 to 4).

## Discussion

Clinical guidelines and risk calculators are the results of rigorous and comprehensive, concerted appraisals of the best available high-quality evidence [11]. Such tools have been elucidated to improve the quality of care by decreasing unnecessary hospital admissions and investigations, as well as identifying patients who require urgent interventions [12,13]. Evidence suggests non-compliance with national guidelines is high and guidelines do not consistently change clinical practice. Lack of adherence to guidelines has led to preventable harm being the third leading cause of death in the United States (US); furthermore, it has resulted in one-third of health care spending on therapies that do not improve patient care, estimated at nearly $1 trillion [10,14-16]. The National Institute for Health and Care Excellence has previously published comprehensive guidelines for the management of UGIB, which include risk assessment scores( the Glasgow-Blatchford bleeding score [17] and the Rockall score [18]), the judicious use of endoscopy, other investigations, and prompt initiation of treatment [7]

The BSG CG 2019 [8] was the product of a rigorous and comprehensive consensus-based review process including a total of 16 individuals representing expertise in gastroenterology, surgery, and radiology. The guideline was developed by the Appraisal of Guidelines for Research & Evaluation (AGREE) methodology [19] and Oakland et al. detailed the process of finalizing recommendations in their article [8]. The BSG CG 2019 [8] proposes the use of calculating Shock index (SI) [20] to characterize the stability of bleeds (SI>1=unstable GIB; SI<1 =stable GIB). The SI indicates the risk of developing hemodynamic shock and is calculated by dividing heart rate (HR) by systolic blood pressure (SBP). Findings reported from NCEPOD [1] later reinforced the utility of SI, reporting that raised SI in LGIB was significantly associated with mortality. The BSG CG 2019 recommends calculating the externally validated Oakland score [21] (based on age, gender, previous LGIB admission, rectal examination findings, HR, SBP, and hemoglobin) in patients with stable GIB (SI<1) to predict severity of LGIB (minor LGIB: Oakland score ≤8; major LGIB: Oakland score >8).

Two key recommendations from BSG CG 2019 for categorizing LGIB included the calculation of SI (SI >1=unstable GIB; SI<1=stable GIB) and Oakland score (minor LGIB: Oakland score ≤8; major LGIB: Oakland score >8). Patients stratified to have minor LGIB as per Oakland score can be safely discharged with outpatient investigations. Our survey found that approximately 75% of respondents either do not calculate SI and Oakland scores or are unsure about them. This subsequently affected overall further decision making and patient management, which later translated into 57.8% of respondents opting for admitting patients with minor unstable bleed.

The national audit carried out by the Association of Coloproctology of Great Britain and Ireland (ACPGBI) and BSG in 2016 revealed that 72.8% of hospitals had access to 24/7 lower GI endoscopy services and 25.9% of patients had inpatient flexible sigmoidoscopy or colonoscopy [8]. We identified only 32.8% (n=21) admit patients with stable major LGIB for colonoscopy and a marginally higher proportion (n=23, 35.9%) admit such patients for CT angiogram; 31.3% of respondents would admit these patients for conservative treatment and outpatient investigations. Although our survey did not specifically look into the availability of 24/7 endoscopy services, the lack of this could be a reason for the small number of respondents who opted for colonoscopy as the first line of investigation for patients with major stable bleeds. Given this, it may be wise for future updates of BSG recommendations to guide centers without immediate access to such facilities. Further, the unnecessary use of CT angiograms exposes patients to significant radiation that increases the risk of cancer [22].

The principal investigator of unstable LGIB is a CT angiogram, which can be conducted at any hospital with facilities for routine CT scanning. In unstable patients, a CT angiogram is recommended over a colonoscopy as it can identify bleeding from both upper and lower GI sources, is easily available, and has a sensitivity of 95% and specificity of almost 100% for detection of the source of acute LGIB [23]. Success rates of IR embolization are reported to be up to 96% [24]. The NCEPOD report recommends that patients with GIB should have access to 24/7 interventional radiology services (on-site or covered by a formal referral network) (1). Interventional radiology has been labeled as a ‘shortage specialty’ due to a lack of trained personnel. A UK workforce census report titled 'Clinical Radiology UK workforce census report', has highlighted this crisis in stark detail [25]. According to this report, there is a 37% shortfall of interventional radiologists, equating to a minimum of 386 additional interventional radiologists required to meet the requirements of an effective 24/7 IR service. A total of 55 (85.6%) respondents in our survey indicated they would resuscitate unstable LGIB with or without urgent CTA, IR embolization, and surgery as indicated. This was the only question where adherence did match significantly with guidelines. In the national audit by ACPGBI and BSG 2016, shocked patients accounted for only 2.3% of all admissions.

Of the 67.2% of respondents who had onsite IR facilities, only 57.4% reported that this was available 24/7. Despite the NCEPOD report in 2015 recommending that there should be 24/7 IR facilities (on-site or covered by a formal network), our survey shows that there is significant variability in the availability of this very important service. Amongst the hospitals which did have on-site IR facilities, only 61% had a definite referral pathway for embolization while 11% were unsure of the same. The survey further identified variation in perceived ease of referring patients for IR embolization at night (median 3 [IQR 2 to 4], Likert scale: 1 being extremely difficult, 5 extremely easy). Greater clarity is therefore needed regarding pathways for escalation and transfer to centers with the availability of such facilities.

Of the respondents, 65.6% agreed that the BSG CG 2019 management algorithm is applicable in their system. Although logistical and technical issues exist in the form of the availability of IR facilities and on-site 24 hours endoscopy, it affects only a very small proportion of patients. The BSG CG 2019 guidelines, in general, do not need alterations in existing hospital setups and can be implemented by increasing awareness and adherence to risk assessment tools and stratification.

There were 5.8 million emergency admissions in England from 2016 to 2017, of which 24% might have been avoided [26]. The NCEPOD found minor LGIB patients (Oakland score <8) had an average hospital length of stay of four days, costing an estimated 1600 pounds per patient (400 pounds per day). There appears to be a significant lack of adherence to the new recommendations from the surgical community we surveyed across the UK and Ireland, which could be due to numerous reasons including lack of awareness, personal preferences, and general reliance upon clinical acumen rather than objective assessment tools. Wider implementation and integration of BSG CG 2019 [8] with Trust (organizations functioning under the remit of the national health service (NHS), delivering medical care within the UK) policies may encourage greater acceptance and adherence to the guidelines.

We acknowledge that there are limitations to this study. Firstly, although there was a mixed geographical distribution amongst the participants, we received a modest number of responses (64). Secondly, we did not explore the reasons for variations in practice nor did we investigate solutions to improve the lack of adherence. We only explored self-reported outcomes and did not correspond such findings with clinical data, which may have resulted in reporting bias. 

## Conclusions

This snapshot survey highlights the significant variations in LGIB management practice within the UK with patchy adherence to guidelines leading to avoidable admissions. These variations probably stem from a lack of proper dissemination of guidelines or logistical difficulties as a result of a lack of staffing and resources. To harmonize the management of LGIB and improve outcomes within the UK, there is an urgent need to facilitate the processes by highlighting this variation in practice through bodies such as the ACPGBI and ASGBI.
